# Adult stem cell transplantation combined with conventional therapy for the treatment of end-stage liver disease: a systematic review and meta-analysis

**DOI:** 10.1186/s13287-021-02625-x

**Published:** 2021-10-30

**Authors:** Chen-Hui Zhu, Dian-Han Zhang, Chen-Wei Zhu, Jing Xu, Chuan-Long Guo, Xiang-Gen Wu, Qi-Long Cao, Guo-Hu Di

**Affiliations:** 1 School of Basic Medicine, Qingdao University, 308 Ningxia Road, Qingdao, 266071 China; 2 College of Chemical Engineering, Qingdao University of Science and Technology, Qingdao, China; 3 Qingdao Haier Biotech Co. Ltd, Qingdao, China

**Keywords:** End-stage liver disease, Stem cell transplantation, Conventional therapy, Adult stem cells, Liver function

## Abstract

End-stage liver disease (ESLD) is characterized by the deterioration of liver function and a subsequent high mortality rate. Studies have investigated the use of adult stem cells to treat ESLD. Here, a systematic review and meta-analysis was conducted to determine the efficacy of a combination therapy with adult stem cell transplantation and traditional medicine for treating ESLD. Four databases—including PubMed, Web of Science, Embase, and Cochrane Library—were investigated for studies published before January 31, 2021. The main outcome indicators were liver function index, model for end-stage liver disease (MELD) scores, and Child‒Turcotte‒Pugh (CTP) scores. Altogether, 1604 articles were retrieved, of which eight met the eligibility criteria; these studies included data for 579 patients with ESLD. Combination of adult stem cell transplantation with conventional medicine significantly improved its efficacy with respect to liver function index, CTP and MELD scores, but this effect gradually decreased over time. Moreover, a single injection of stem cells was more effective than two injections with respect to MELD and CTP scores and total bilirubin (TBIL) and albumin (ALB) levels, with no significant difference in aspartate aminotransferase (AST) and alanine aminotransferase (ALT) levels. With respect to the TBIL levels, patients receiving mononuclear cells (MNCs) experienced a significantly greater therapeutic effect—starting from twenty-four weeks after the treatment—whereas with respect to ALB levels, CD34^+^ autologous peripheral blood stem cells (CD34^+^ APBSCs) and MNCs had similar therapeutic effects. Severe complications associated with adult stem cell treatment were not observed. Although the benefits of combination therapy with respect to improving liver function were slightly better than those of the traditional treatment alone, they gradually decreased over time.

*Systematic review registration*: PROSPERO registration number: CRD42021238576.

**Supplementary Information:**

The online version contains supplementary material available at 10.1186/s13287-021-02625-x.

## Introduction

The natural course of cirrhosis is characterized by the progression from an early asymptomatic phase—termed “compensated cirrhosis”—to the development of portal hypertension and complications, with or without liver dysfunction, termed “decompensated cirrhosis” or “end-stage liver disease” (ESLD) [1]. The decompensated stage can be further subclassified into a more severe stage, characterized by the development of recurrent variceal hemorrhage, refractory ascites, hyponatremia, and/or hepatorenal syndrome [2, 3]. Patients with ESLD who are hospitalized in the intensive care unit (ICU) have a mortality rate of 40–80% [4–8]. Currently, liver transplantation is the only effective treatment for ESLD [9]. However, the failure of liver transplantation will lead to further progressive fibrosis that will impede liver regeneration and cause irreversible cirrhosis [10, 11]. As the demand for donor liver substantially exceeds its availability, an increasing number of patients with ESLD are dying while awaiting liver transplantation; thus, there is a need to develop strategies for alternative therapy [12, 13].

Since the first human bone marrow transplantation (BMT) and the discovery of hematopoietic stem cell/progenitors (HSC/Ps) via clonal assays, adult stem cells originating from various tissues have been researched [14, 15]. Adult stem cells, such as CD34^+^ peripheral blood stem cells (CD34^+^ PBSCs), bone marrow-derived mesenchymal stromal cells (BMMSCs), and bone marrow mononuclear cells (BMMNCs), have excellent potential as regenerative medicine in different therapeutic applications [16–22]. In addition, there are many examples of the successful clinical usage of adult stem cells. Adult hematopoietic stem cells can repopulate the bone marrow of leukemia patients after transplantation and produce all blood cell lineages, and skin stem cells can be used to treat patients with severe burns [23].

Animal studies show that adult stem cells exert substantial protective effects against ESLD and improve the associated survival rate [24–27]. It is generally believed that cytokines, chemokines, and growth factors—secreted by adult stem cells—effectively reduce inflammation and inhibit hepatocyte apoptosis in both acute and chronic liver injury models [28–31]. Although multiple clinical trials on various sources of adult stem cells for treating ESLD have revealed positive results [32–37], some have reported unsatisfactory efficacy or no significant improvement [38]. As the commonly used therapies remain ineffective, it is important to search for alternative therapies for reducing symptoms and improving the quality of life in patients with ESLD. Therefore, the purpose of this meta-analysis is to evaluate the efficacy of a combinatorial therapy of adult stem cells and traditional supportive therapy in patients with ESLD and to find the best possible treatment.

## Methods

This review protocol was registered with PROSPERO (registration number: CRD42021238576). All procedures in this study followed the Preferred Reporting Items for Systematic Reviews and Meta-Analyses (PRISMA) guidelines [39].

### Data sources and search strategy

A systematic search was performed for relevant publications in PubMed, Embase, Web of Science, and the Cochrane Library databases through January 31, 2021. The retrieval strategy was determined according to the principle of patient-intervention-comparison-outcome-study design for exploring the efficacy of adult stem cells in the treatment of ESLD. Literature search strategies typically use a combination of terms from Medical Subject Headings (MeSH) and free-text keywords. The search terms were “end-stage liver disease” OR “chronic liver failure” OR “chronic liver failures” OR “failure, chronic liver” OR “failures, chronic liver” OR “liver failures, chronic” OR “liver failure, chronic” AND “stem cells” OR “cell, stem” OR “stem cell” OR “cell, progenitor” OR “progenitor cell” OR “cell, mother” OR “mother cell” OR “colony-forming unit” OR “colony-forming units” OR “cells, stem” OR “progenitor cells” OR “cells, progenitor” OR “mother cells” OR “cells, mother” OR “mesenchymal stromal cell” OR “adult stem cell.”

Moreover, to obtain more potential literature, a manual search was carried out to retrieve and screen the relevant reference lists of reviews, systematic reviews, meta-analyses, and included literature. All references were imported into the reference management software, EndNote, version X9.

### Screening and selection of studies

The results of the literature search were downloaded into EndNote, and duplicate articles were removed. Two reviewers (Zhu CH and Zhang DH) independently screened the studies in two rounds, first with abstracts alone and then with full-text articles with eligible abstracts. All clinical trials were required to meet the following inclusion criteria:

- Clinical study
- Adult stem cells were used for ESLD treatment
- The experimental group received both conventional medical intervention and adult stem cells, whereas the control group was prescribed a conventional medication regimen
- All patients received a definitive diagnosis of ESLD
- Availability of clinical outcomes [for example, alanine aminotransferase (ALT), aspartate aminotransferase (AST), total bilirubin (TBIL), and albumin (ALB) levels; prothrombin time (PT); prothrombin time activity (PTA); and Child‒Turcotte‒Pugh (CTP) and model for end-stage liver disease (MELD) scores]
- Patients were over 15 years of age
- Studies with at least one-month follow-up after cell transplantation
- The routes of stem cell transplantation were not restricted

Studies on populations that satisfied a mixture of criteria defined under inclusion and exclusion criteria were included if the data for ESLD were presented separately.

Clinical trials were excluded if they met any of the following criteria:

- Repeatedly published data
- Animal-based, review articles, letters, editorials, abstracts, expert opinions, comments, basic science research, or case reports
- Unavailability of full text or adequate information (data) related to the pre-specified outcome
- Overlapping or duplicated data among two or more studies by the same team (only the study with more complete/recent data or the one with the longer follow-up period was included)
- Studies that involved patients with coexisting liver tumors (inability to ascertain the effect of stem cells on tumor pathogenesis), kidney or heart failure, human immunodeficiency virus infection, and portal vein thrombosis, or pregnant patients
- Not published in English.

### Data extraction and quality appraisal

For efficient data collection, one researcher extracted the data and another researcher independently checked the data extraction strategies for accuracy and completeness. The following were the types of data extracted: first authors, publication year, country, affiliations, number of patients, patient characteristics (age, sex), therapeutic regimen, study design, cell source, disease etiology, route of injection, timing of injection, and the duration of follow-up.

The main outcome indicators were TBIL, ALB, AST, and ALT levels, PT, PTA, and MELD, and CTP scores. Two authors independently assessed the quality of the eight included studies, using the Cochrane Handbook for Systematic Reviews of Interventions criteria, and disagreements between the two authors were resolved with a discussion until a consensus was reached.

### Statistical analysis

A meta-analysis of the eight clinical trials was conducted to evaluate the efficacy of adult stem cells in the treatment of ESLD. Standardized mean difference (SMD)/weighted mean difference (WMD) and 95% confidence interval (CI) were evaluated for liver function parameters, including the MELD score and levels of ALT and ALB. The Q test (Chi-square test) and *I*^2^ statistic were used to evaluate heterogeneity among the eligible studies. When *P* < 0.1 or *I*^2^ > 50%, a high level of heterogeneity between the studies was assumed and a random-effect model was adopted; when no significant heterogeneity was identified between studies, a fixed-effect model was used. Sensitivity analysis was performed to determine the potential source of heterogeneity. Funnel plots were produced to evaluate the possible publication bias. All data analyses were performed using the RevMan (version 5.4.1) software.

## Results

### Screening and characteristics of the included studies

**Fig. 1:**
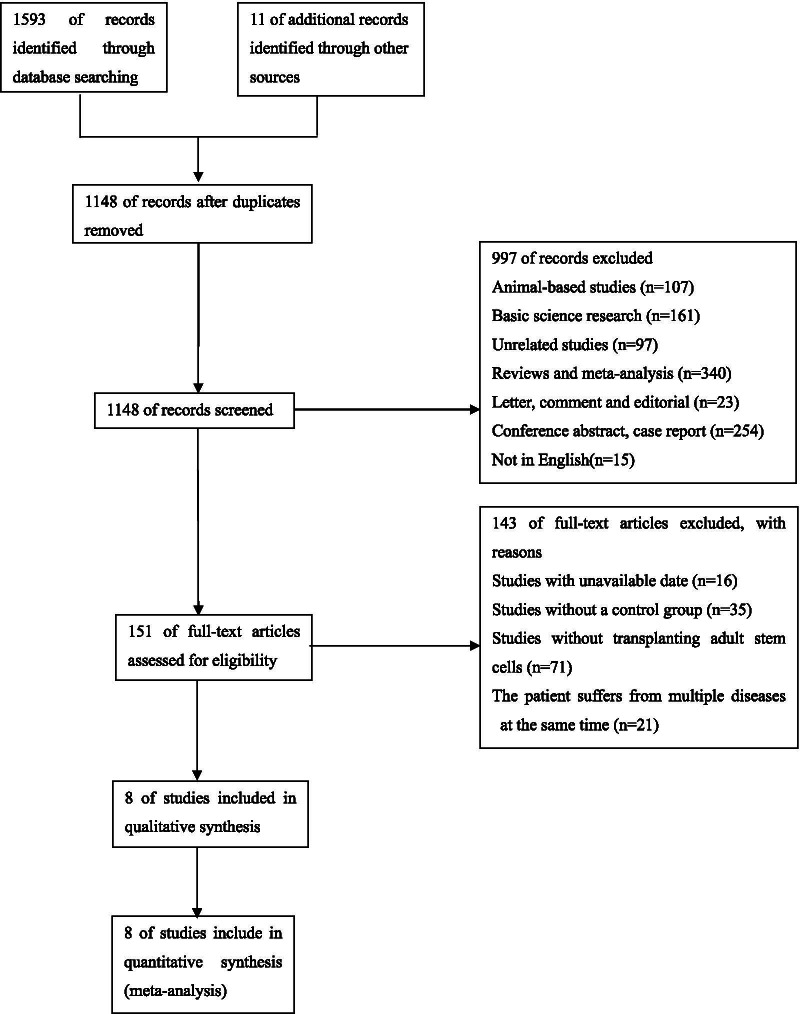
Flow chart describing the selection of studies for the systematic review and meta-analysis of comparing the efficacy of adult stem cell combination therapy with standardized medical treatment in patients with ESLD, showing the number of studies excluded at each step, as well as the reasons for exclusion from the systematic review and meta-analysis. A total of 1593 references were retrieved from the four database, and 11 articles were obtained by manual retrieval. Of these 1604 studies, eight articles met the inclusion criteria for the final analysis after full-text evaluation

Based on the selected search terms, a total of 1593 references were retrieved from the database and 11 articles were manually retrieved. After full-text evaluation of 1604 studies, 8 met the inclusion criteria for final analysis (Fig. 1) [40–47].

These eight articles—that included data for 579 patients—were analyzed. In all eight studies, stem cell species identification and activity assessment had been carried out. Seven articles (87.5%) were based on studies conducted in Asia (six from China [41–46], one article was from Korea [47]), and one (12.5%) was from Egypt [40]. Etiology-based analysis of the studies revealed the following: patients with only severe alcoholic hepatitis (*n* = 1 study) [47]; patients with ESLD due to hepatitis C virus (HCV) infection (*n* = 1 study) [40]; patients with only hepatitis B virus (HBV)‒associated ESLD (*n* = 5 studies) [42–46]; ESLD patients with hepatitis B virus (HBV) or alcoholic hepatitis (*n* = 1 study) [41]. Analysis of the frequency of stem cell injections revealed the following: single stem cell injections (*n* = 6 studies) [40–43, 45, 46]; two spaced stem cell injections (*n* = 1 study) [44]; a mix of single and two stem cell injections (*n* = 1 study) (Table 1) [47].

**Table 1:** Clinical information from the eligible trials in the meta-analysis

First authors/published year	Country	No. of patients		Therapeutic regimen		Cell source	Cell dosage
		Con	Exp	Con	Exp		
Cai et al. 2015	China	28	23	Conventional medical treatment	Conventional medical treatment and G-CSF CD34^+^ APBSC transplantation	G-CSF CD34^+^ APBSC	(2.0–4.0) × 10^7
Liu et al. 2014	China	37	40	Lamivudine and adefovir dipivoxil	G-CSF ABMMSC transplantation in combination with lamivudine and adefovir dipivoxil	G-CSF ABMMSC	(1.6–3.2) × 10^11
Bai et al. 2014	China	15	32	Routine medical treatment	Treated with BM-MNCs plus a conventional internal medicine regimen	BM-MNCs	NA
Al Tayeb et al. 2015	Egypt	10	10	Conventional therapy	Conventional therapy and the G-CSF PB-MNCs transplantation	G-CSF PB-MNCs	NA
Deng et al. 2015	China	35	33	Conventional medical therapy	Conventional medical therapy and G-CSF CD34^+^ APBSC transplantation	G-CSF CD34^+^ APBSC	(2.0–4.0) × 10^7
Fang et al. 2018	China	53	50	Normal medical treatments	Normal medical treatments with the transplantation of hUCMSCs at twice	hUCMSCs	(4.0–4.5) × 10^8
Peng et al. 2011	China	105	53	Conventional medical therapy	Conventional medical therapy and ABMMSC transplantation	ABMMSC	1.0 × 10^7
Suk et al. 2016	Korea	18	Exp1:18 Exp2:19	Treatment-guideline for AC	Exp1: treatment-guideline for AC and one-time ABMMSC transplantation Exp2: treatment-guideline for AC and two-time ABMMSC transplantation	ABMMSC	5.0 × 10^7

First authors/published year	Age (mean ± SD) (years)		Sex (M/F)		Study design	Disease etiology		Injection route	Times of injection	Length of follow-up (months)
	Con	Exp	Con	Exp		Con	Exp			
Cai et al. 2015	50.82 ± 7.98	51.52 ± 10.30	17/11	14/9	nRCT	HBV	HBV	Hepatic artery	Single	12
Liu et al. 2014	50.4 ± 8.5	51.6 ± 9.2	33/4	37/3	RCT	HBV	HBV	Left and right hepatic arteries	Single	1
Bai et al. 2014	47.4 ± 11.1	46.4 ± 11.6	9/6	20/12	nRCT	HBV:13 AC: 2	HBV:30 AC: 2	Hepatic artery	Single	24
Al Tayeb et al. 2015	49.20 ± 3.27	49.20 ± 3.27	10/0	10/0	nRCT	HCV	HCV	Hepatic artery	Single	6
Deng et al. 2015	50.20 ± 10.64	49.48 ± 11.07	12/23	20/13	RCT	HBV	HBV	Hepatic artery	Single	12
Fang et al. 2018	46.55 ± 9.08	46.62 ± 10.17	49/4	45/5	RCT	HBV	HBV	Vein	Twice	13
Peng et al. 2011	42.22 ± 11.37	42.19 ± 10.80	99/6	50/3	nRCT	HBV	HBV	Proper hepatic artery	Single	48
Suk et al. 2016	53.7 ± 8.2	Exp1:53.1 ± 8.7 Exp2:54.4 ± 7.9	17/1	Exp1:15/3 Exp2:17/2	RCT	AC	AC	Hepatic artery	Exp1:single Exp2: twice	12

APBSCs, autologous peripheral blood stem cells; BM-MNCs, bone marrow mononuclear cells; hUCMSCs, human umbilical cord mesenchymal stem cells; ABMMSC, autologous bone marrow mesenchymal stromal cells; PB-MNCs, peripheral blood mononuclear cells; AC, alcoholic cirrhosis; nRCT, non-randomized control trial; RCT, randomized control trial; HBV, hepatitis B virus; HCV, hepatitis C virus; NA, not available; G-CSF, granulocyte colony-stimulating factor

### Methodological quality and risk of bias in the included studies

**Fig. 2:**
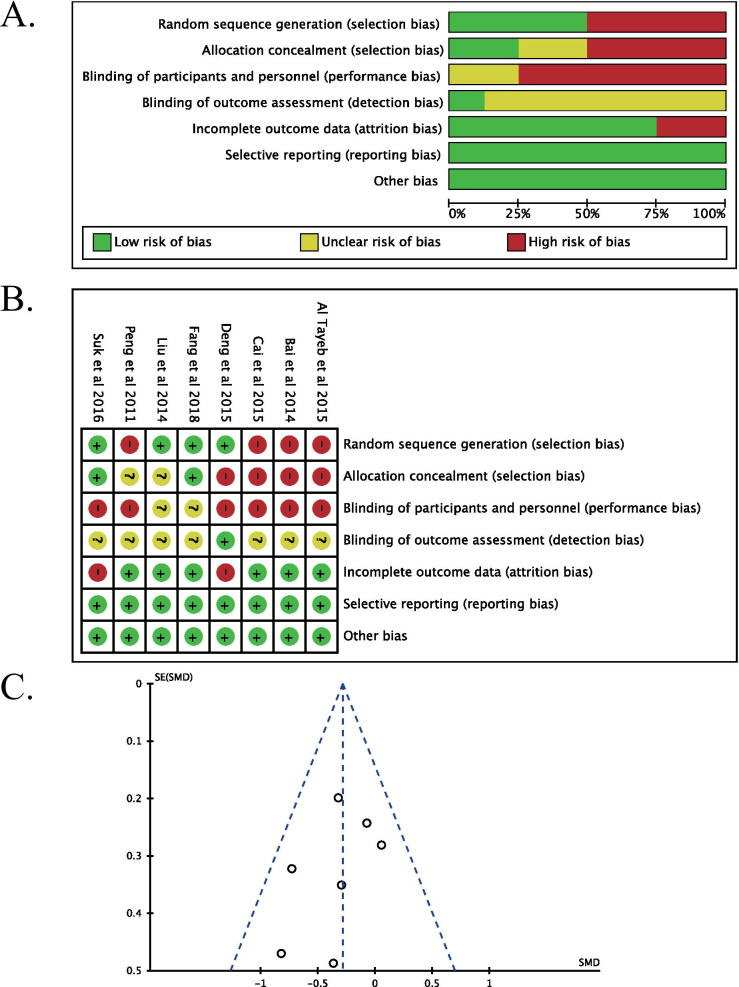
Assessment of risk of bias based on the evaluation domains listed in the Cochrane Handbook for Systematic Reviews of Interventions criteria: **A** Risk of bias graph. A plot of the distribution of review authors’ judgments across studies for each risk of bias item presented as percentages. Note: Each color represents a different level of bias: red for high risk, green for low risk and yellow for unclear risk of bias. **B** Risk of bias summary: a review of the authors’ judgments about each risk of bias domain for each included study. *Note*: Each symbol represents a different level of bias: (?) = unclear risk of bias, (+) = low risk of bias, (−) = high risk of bias. **C** Funnel plot of publication bias analysis for published studies. Note: The circles represent individual studies included in the meta-analysis

The quality evaluation of the included studies is shown in Fig. 2. The studies selected for this review included eight prospective interventional studies (*n* = 579 patients), including four randomized controlled trials (*n* = 303 patients) [43–45, 47], four non-randomized controlled trials (*n* = 276 patients) [40–42, 46], and one study clearly stating the blindedness of the outcome evaluators [43]. Two studies were deemed to have a high risk of attrition bias as the participants were lost to follow-up [43, 47]. A symmetric funnel plot that illustrates the absence of publication bias is shown in Fig. 2. All levels of deviation are presented as a RevMan risk-of-bias plot.

### Assessment of therapeutic effects

#### Effectiveness of the combination therapy with respect to TBIL levels

**Fig. 3:**
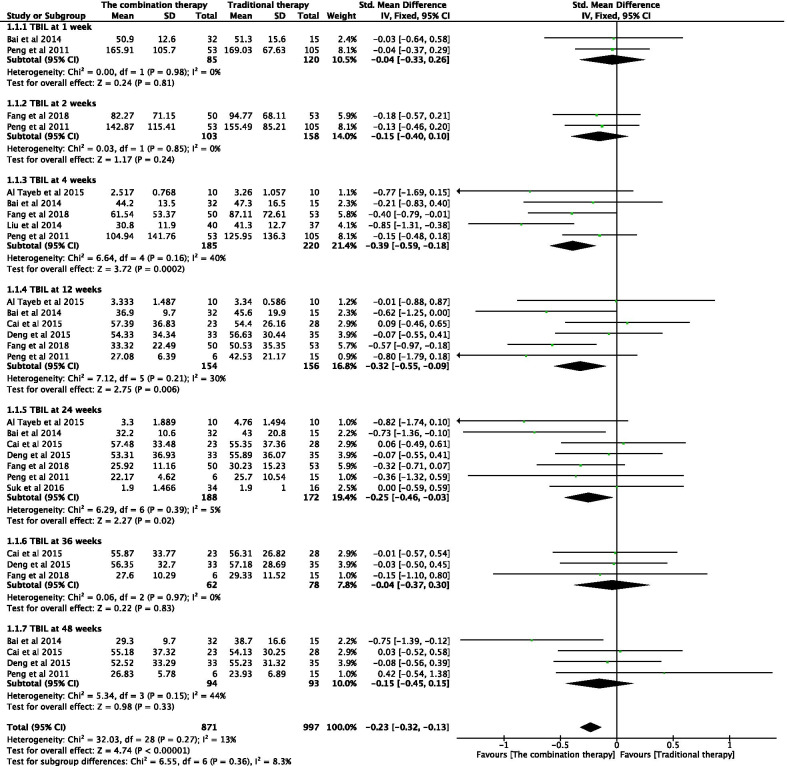
Forest plot of TBIL at different time points with the combination therapy compared with traditional therapy. Compared with the traditional therapy group, TBIL in the combination therapy group was nonsignificantly lower at 1, 2, 36, and 48 weeks (*P* > 0.05), significantly lower at 4, 12, and 24 weeks (*P* < 0.05). No significant heterogeneity was observed in any of the seven groups, and a fixed-effects model was used for statistical analysis. In the plane rectangular coordinate system, the forest plot takes a vertical invalid line (scale of abscissa is 0) as the center, describes the effect quantity and 95% CI of each study by using multiple line segments parallel to the horizontal axis, and describes the effect quantity and confidence interval of multiple studies by using a diamond. TBIL, total bilirubin; CI, confidence interval; SMD, standardized mean difference

The TBIL levels of patients were independently tested at 1, 2, 4, 12, 24, 36, and 48 weeks after the transplantation of adult stem cells (Fig. 3). The combination of adult stem cells with conventional medical treatment was associated with moderate reduction in the TBIL levels at 4 weeks (SMD − 0.39; 95% CI − 0.59 to − 0.18; *P* = 0.0002), 12 weeks (SMD − 0.32; 95% CI − 0.55, − 0.09; *P* = 0.006), and 24 weeks post-transplantation (SMD − 0.25; 95% CI − 0.46, − 0.03; *P* = 0.02) (Fig. 3). No significant difference was observed at any other time point. No significant heterogeneity was observed in any of the seven groups, and a fixed-effects model was used for statistical analysis.

#### Effectiveness of the combination therapy with respect to ALB levels

**Fig. 4:**
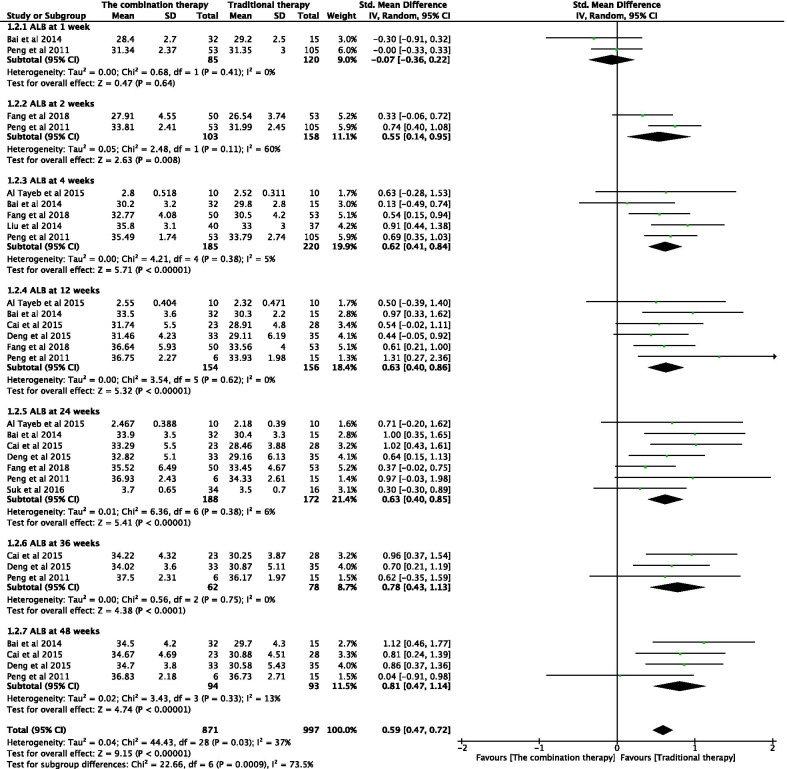
Forest plot of ALB at different time points with the combination therapy compared with traditional therapy. Compared with the traditional therapy group, ALB in the combination therapy group was nonsignificantly higher at 1 week (*P* > 0.05), significantly higher at 2, 4, 12, 24, 36, and 48 weeks (*P* < 0.05). Since significant heterogeneity was observed in one group (*I*^2^ = 60%), a random-effects model was used. In the plane rectangular coordinate system, the forest plot takes a vertical invalid line (scale of abscissa is 0) as the center, describes the effect quantity and 95% CI of each study by using multiple line segments parallel to the horizontal axis, and describes the effect quantity and confidence interval of multiple studies by using a diamond. ALB, albumin; CI, confidence interval; SMD, standardized mean difference

The ALB levels of patients were independently tested at 1, 2, 4, 12, 24, 36, and 48 weeks after the transplantation of adult stem cells (Fig. 4). Combination of adult stem cells with conventional medical treatment was associated with significantly increased ALB levels at 2 weeks (SMD 0.55; 95% CI 0.14–0.95; *P* = 0.008), 4 weeks (SMD 0.62; 95% CI 0.41–0.84; *P* < 0.00001), 12 weeks (SMD 0.63; 95% CI 0.40–0.86; *P* < 0.00001), 24 weeks (SMD 0.63; 95% CI 0.40–0.85; *P* < 0.00001), 36 weeks (SMD 0.78; 95% CI 0.43–1.13; *P* < 0.0001), and 48 weeks post-transplantation (SMD 0.81; 95% CI 0.47–1.14; *P* < 0.00001). No significant difference was observed at any other time point. However, significant heterogeneity was observed in one group (*I*^2^ = 60%). A random-effects model—that accounts for statistical heterogeneity between the studies and provides a more conservative estimate of the significance than a fixed-effects model—was used. Sensitivity analysis was conducted by eliminating any single study, and no significant changes were observed on combining the results, indicating that the results of the study were relatively stable and reliable.

#### Effectiveness of the combination therapy with respect to ALT levels

**Fig. 5:**
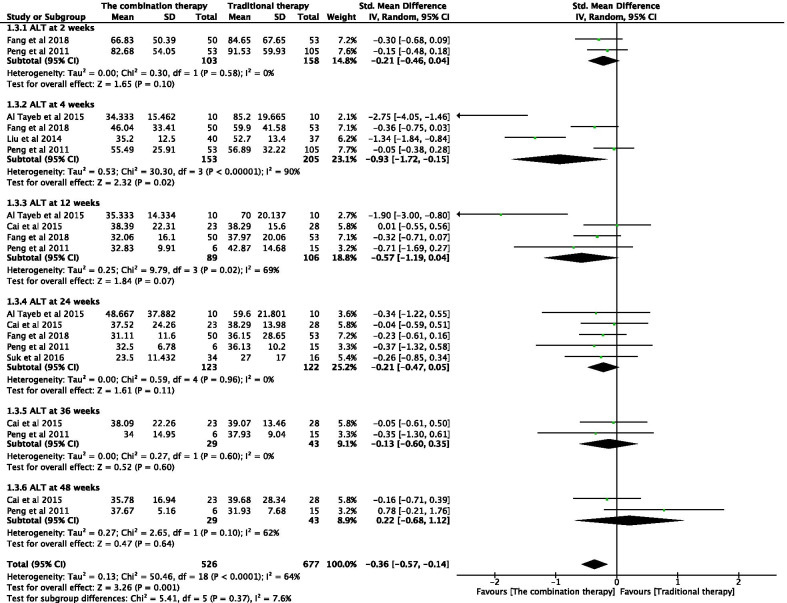
Forest plot of ALT at different time points with the combination therapy compared with traditional therapy. Compared with the traditional therapy group, ALT in the combination therapy group was nonsignificantly lower at 2, 12, 24, 36, and 48 weeks (*P* > 0.05), significantly lower at 4 weeks (*P* < 0.05). Since significant heterogeneity was observed among the three groups (*I*^2^ = 90%, 69%, and 62%), a random-effects model was used. In the plane rectangular coordinate system, the forest plot takes a vertical invalid line (scale of abscissa is 0) as the center, describes the effect quantity and 95% CI of each study by using multiple line segments parallel to the horizontal axis, and describes the effect quantity and confidence interval of multiple studies by using a diamond. ALT, alanine aminotransferase; CI, confidence interval; SMD, standardized mean difference

The ALT levels of patients were independently tested at 2, 4, 12, 24, 36, and 48 weeks after the transplantation of adult stem cells (Fig. 5). A combination of adult stem cells with conventional medical treatment was associated with significantly lower ALT level at four weeks post-transplantation (SMD − 0.93; 95% CI − 1.72 to − 0.15; *P* = 0.02) (Fig. 5); however, these findings were highly inconsistent. Significant heterogeneity was observed among the three groups (*I*^2^ = 90%, 69%, and 62%). To reach a conservative estimate, a random-effects model was used to account for the highly significant inter-study heterogeneity. For week 4 and 12 measurements, sensitivity analysis was conducted by eliminating any single study. No significant changes were observed after combining the results, indicating that the results of the study were relatively stable and reliable. However, in the week 12 measurement, two studies showed conflicting results considering that the extended follow-up resulted in a significant difference between the two groups. No significant difference was observed at any other time point.

#### Effectiveness of the combination therapy with respect to AST levels

**Fig. 6:**
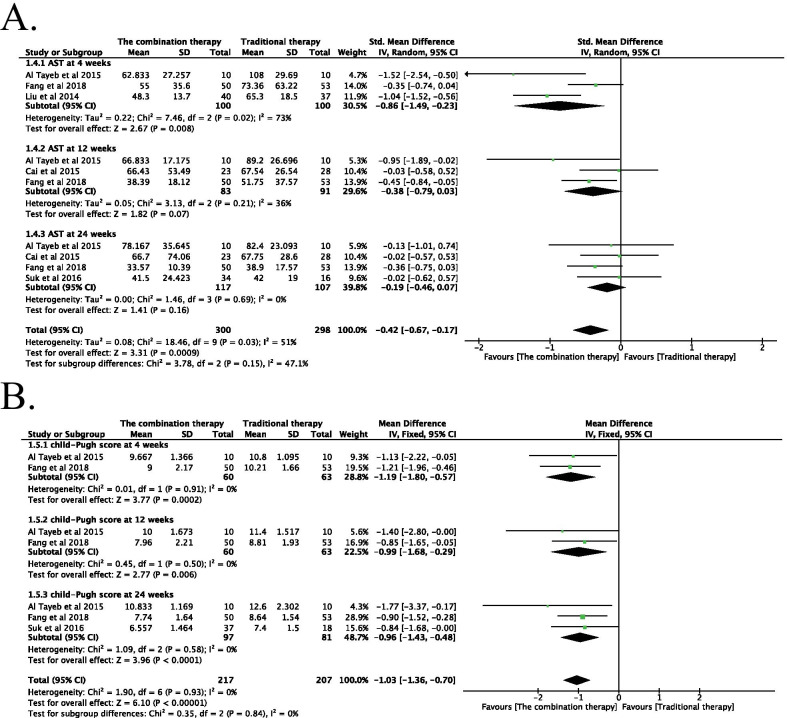
**A** Forest plot of AST at different time points with the combination therapy compared with traditional therapy. Compared with the traditional therapy group, AST in the combination therapy group was nonsignificantly lower at 12, and 24 weeks (*P* > 0.05), significantly lower at 4 weeks (*P* < 0.05). Since significant heterogeneity was observed in one group (*I*^2^ = 73%), a random-effects model was used. **B** Forest plot of CTP at different time points with the combination therapy compared with traditional therapy. Compared with the traditional therapy group, CTP in the combination therapy group was significantly lower at 4, 12, and 24 weeks (*P* < 0.05). No significant heterogeneity was observed; therefore, a fixed-effects model was used for statistical analysis. In the plane rectangular coordinate system, the forest plot takes a vertical invalid line (scale of abscissa is 0) as the center, describes the effect quantity and 95% CI of each study by using multiple line segments parallel to the horizontal axis, and describes the effect quantity and confidence interval of multiple studies by using a diamond. AST, aspartate aminotransferase; CTP, Child–Pugh score; CI, confidence interval. SMD, standardized mean difference. WMD, weighted mean difference

The AST levels of patients were independently tested at 4, 12, and 24 weeks after the transplantation of adult stem cells (Fig. 6). The combination of adult stem cells with conventional medical treatment was associated with the significant lowering of AST level at 4 weeks post-transplantation (SMD − 0.86; 95% CI − 1.49 to − 0.23; *P* = 0.008) (Fig. 6). No significant difference was observed at any other time point. However, significant heterogeneity was observed in one group (I^2^ = 73%). A random-effects model was used, and the results of the sensitivity analysis were the same as those obtained for ALB.

#### Effectiveness of the combination therapy with respect to the Child‒Turcotte‒Pugh Score

The CTP scores of the patients were independently tested at 4, 12, and 24 weeks after the transplantation of adult stem cells (Fig. 6). The combination of adult stem cells with conventional medical treatment was associated with a significantly lower CTP score at 4 weeks (WMD − 1.19; 95% CI − 1.80 to − 0.57; *P* = 0.0002), 12 weeks (WMD − 0.99; 95% CI − 1.68 to − 0.29; *P* = 0.006), and 24 weeks post-transplantation (WMD − 0.96; 95% CI − 1.43 to − 0.48; *P* < 0.0001). No significant difference was observed at any other time point. No significant heterogeneity was observed in any of the seven groups, and a fixed-effects model was used for statistical analysis.

#### Effectiveness of the combination therapy with respect to the MELD Score

**Fig. 7:**
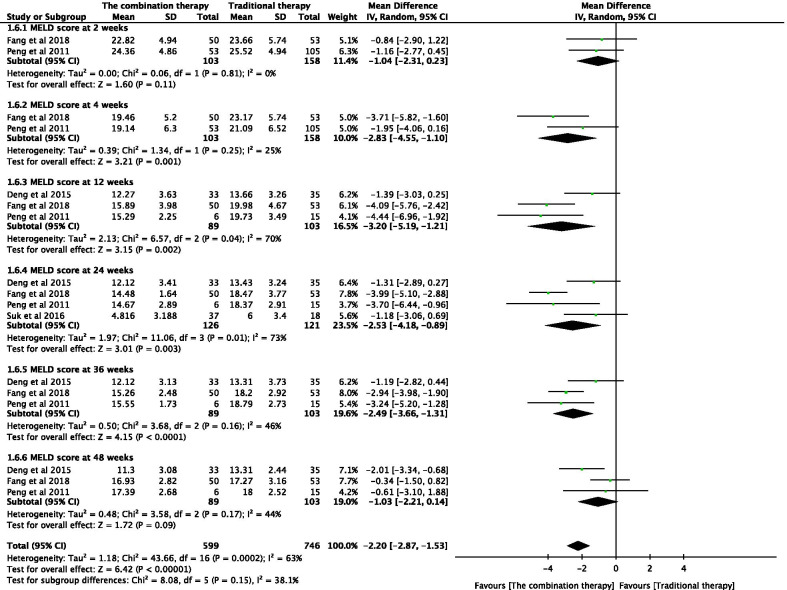
Forest plot of MELD at different time points with the combination therapy compared with traditional therapy. Compared with the traditional therapy group, MELD in the combination therapy group was nonsignificantly lower at 2, and 48 weeks (*P* > 0.05), significantly lower at 4, 12, 24, and 36 weeks (*P* < 0.05). Since significant heterogeneity was observed between the two groups (*I*^2^ = 70% and 73%), a random-effects model was used. In the plane rectangular coordinate system, the forest plot takes a vertical invalid line (scale of abscissa is 0) as the center, describes the effect quantity and 95% CI of each study by using multiple line segments parallel to the horizontal axis, and describes the effect quantity and confidence interval of multiple studies by using a diamond. MELD, Model for end-stage liver disease; CI, confidence interval. WMD, weighted mean difference

The MELD scores of the patients were independently tested at 2, 4, 12, 24, 36, and 48 weeks after the transplantation of adult stem cells (Fig. 7). Combination of adult stem cells with conventional medical treatment was associated with significantly lower MELD scores at 4 weeks (WMD − 2.83; 95% CI − 4.55 to − 1.10; *P* = 0.001), 12 weeks (WMD − 3.20; 95% CI − 5.19 to − 1.21; *P* = 0.002), 24 weeks (WMD − 2.53; 95% CI − 4.18 to − 0.89; *P* = 0.003), and 36 weeks post-transplantation (WMD − 2.49; 95% CI − 3.66 to − 1.31; *P* < 0.0001). No significant difference was observed at any other time point. However, significant heterogeneity was observed between the two groups (*I*^2^ = 70% and 73%). A random-effects model was used and sensitivity analysis was conducted by eliminating any single study. No significant changes were observed after combining the results, indicating that the results of the study were relatively stable and reliable.

#### Effectiveness of the combination therapy with respect to PTA

The PTA of patients was independently tested at 12, 24, 36, and 48 weeks after the transplantation of adult stem cells (Additional file 1: Figure S1). The combination of adult stem cells with conventional medical treatment was associated with significantly increased PTA at 12 (WMD 5.61; 95% CI 2.41–8.80; *P* = 0.0006), 24 weeks (WMD 8.01; 95% CI 4.87–11.14; *P* < 0.00001), 36 weeks (WMD 6.08; 95% CI 3.43–8.73; *P* < 0.00001), and 48 weeks post-transplantation (WMD 6.94; 95% CI 4.16–9.73; *P* < 0.00001) (Additional file 1: Figure S1). However, no significant difference was observed at any other time point. No significant heterogeneity was observed in any of the seven groups, and a fixed-effects model was used for statistical analysis.

#### Effectiveness of the combination therapy with respect to PT

The PT of patients was independently tested at 1, 2, 4, 12, 24, and 48 weeks after the transplantation of adult stem cells (Additional file 1: Figure S2). The combination of adult stem cells with conventional medical treatment was associated with significantly lower PT level at 2 weeks (WMD − 1.47; 95% CI − 2.26 to − 0.68; *P* = 0.0003), 4 weeks (WMD − 2.27; 95% CI − 4.00 to − 0.55; *P* = 0.010), 12 weeks (WMD − 2.62; 95% CI − 3.77 to − 1.48; *P* < 0.00001), 24 weeks (WMD − 1.26; 95% CI − 2.06 to − 0.46; *P* = 0.002), and 48 weeks post-transplantation (WMD − 1.48; 95% CI − 2.90 to − 0.06; *P* = 0.04). No significant difference was observed at any other time point. However, significant heterogeneity was observed in one group (*I*^2^ = 87%). To reach a conservative estimate, a random-effects model was used to account for the highly significant inter-study heterogeneity. Sensitivity analysis inferred no significant changes in the results, indicating their stability and reliability.

### Subgroup analysis

After extracting data from the eight included studies, two subgroup analysis were performed for factors that were thought to influence the outcomes. With respect to the number of injection cycles of stem cells, six studies [40–43, 45, 46] focused on single injection, one study [44] on two injections, and one study [47] focused on the effects of both single and two injections. Regarding the species of adult stem cells, CD34^+^ APBSCs were transplanted in two studies [42, 43], MNCs were transplanted in two studies [40, 41], and autologous BMMSCs were used in two studies [46, 47]. Four studies [40, 42, 43, 45] mobilized stem cells using granulocyte colony-stimulating factor (G-CSF) followed by their collection, while the remaining four studies [41, 46–48] collected stem cells directly. Subgroup analyses were performed based on the number of stem cell injection cycles, the species of adult stem cells, and on usage of G-CSF. Surprisingly, the TBIL (Additional file 1: Figure S3), ALB levels (Additional file 1: Figure S3), and MELD scores (Additional file 1: Figure S4) differed between patients receiving single and double injection of stem cells (TBIL: single injection—SMD − 0.27; 95% CI − 0.53 to − 0.01; *P* = 0.04; two injections—SMD − 0.19; 95% CI − 0.53 to 0.15; *P* = 0.26; ALB: single injection—SMD 0.78; 95% CI 0.51–1.05; *P* < 0.00001; two injections—SMD 0.31; 95% CI − 0.03 to 0.65; *P* = 0.07; MELD: single injection—WMD − 1.90; 95% CI − 3.09 to − 0.71; *P* = 0.002; two injections—WMD − 2.45; 95% CI − 5.76 to 0.86; *P* = 0.15). However, the levels of ALT (Additional file 1: Figure S3) and AST (Additional file 1: Figure S4) showed no significant difference between the experimental and the control groups (ALT: single injection—SMD − 0.18; 95% CI − 0.54 to 0.18; *P* = 0.32; two injections—SMD − 0.23; 95% CI − 0.57 0.10; *P* = 0.17; AST: single injection—SMD − 0.09; 95% CI − 0.47 to 0.30; *P* = 0.65; two injections—SMD − 0.26; 95% CI − 0.59, 0.08; *P* = 0.14). The CTP score (Additional file 1: Figure S4) showed no significant difference between the two groups (single injection—SMD − 1.26; 95% CI − 2.06 to − 0.47; *P* = 0.002; two injections—SMD − 0.82; 95% CI − 1.34 to − 0.29; *P* = 0.002). With respect to the association between the species of adult stem cells and efficacy, the TBIL level (Additional file 1: Figure S5) was found to be considerably decreased in studies involving transplantation with MNCs as compared to that in other studies (CD34 + APBSC—SMD − 0.02; 95% CI − 0.38 to 0.35; *P* = 0.93; ABMMSC—SMD − 0.10; 95% CI − 0.61 to 0.40; *P* = 0.69; MNCs—SMD − 0.76; 95% CI − 1.28 to − 0.24; *P* = 0.004). The ALB level (Additional file 1: Figure S5) in patients who received CD34 + APBSCs or MNCs significantly differed from that in patients who had received ABMMSCs (CD34 + APBSC—SMD 0.79; 95% CI 0.42–1.17; *P* < 0.0001; ABMMSC—SMD 0.47; 95% CI − 0.04 to 0.99; *P* = 0.07; MNCs—SMD 0.90; 95% CI 0.37–1.43; *P* = 0.0008). However, patients receiving CD34 + APBSCs or MNCs had very similar ALB levels. With respect to the relationship between the use of G-CSF and the effects of therapy, the TBIL level (Additional file 1: Fgure S6) in patients receiving directly collected stem cells significantly differed from that in patients receiving G-CSF mobilized stem cells (12 weeks: G-CSF—SMD − 0.00; 95% CI − 0.33 to 0.33; *P* = 0.99; non-G-CSF—SMD: − 0.61; 95% CI − 0.93 to − 0.29; *P* = 0.0002; 24 weeks: G-CSF—SMD: − 0.12; 95% CI − 0.46 to 0.21; *P* = 0.48; non-G-CSF—SMD: − 0.33; 95% CI − 0.61 to − 0.06; *P* = 0.02). Similar conclusions were observed for ALB (Additional file 1: Figure S6) and ALT (Additional file 1: Figure S7) measurements at week 12 post-transplantation (ALB: 12 weeks: G-CSF—SMD 0.48; 95% CI 0.15–0.82; *P* = 0.005; non-G-CSF—SMD 0.76; 95% CI 0.44–1.09; *P* < 0.00001; ALT: 12 weeks: G-CSF—SMD − 0.89; 95% CI − 2.75 to 0.98; *P* = 0.35; non-G-CSF—SMD: − 0.37; 95% CI − 0.74 to − 0.01; *P* = 0.04). However, for the levels of ALB (Additional file 1: Figure S6), ALT (Additional file 1: Figure S7), and AST (Additional file 1: Figure S7), no significant differences were recorded between the two groups (ALB: 24 weeks: G-CSF—SMD: 0.78; 95% CI 0.43–1.13; *P* < 0.0001; non-G-CSF—SMD: 0.52; 95% CI 0.24–0.80; *P* = 0.0003; ALT: 24 weeks: G-CSF—SMD − 0.12; 95% CI − 0.59 to 0.34; *P* = 0.61; non-G-CSF—SMD − 0.25; 95% CI − 0.56 to 0.06; *P* = 0.11; AST: 24 weeks: G-CSF—SMD − 0.05; 95% CI − 0.52 to 0.42; *P* = 0.83; non-G-CSF—SMD − 0.26; 95% CI − 0.59 to 0.06; *P* = 0.12).

### Sensitivity analysis

The geographical distribution of these eight studies revealed that seven studies were conducted in Asia [41–47] and one was conducted in Africa [40]. The African study was excluded to avoid any interference of ethnicity, and on combining the data from the remaining seven studies, no significant difference was observed in data sensitivity.

### Adverse effect and complication

Although Bai et al. [41] found that the incidences of spontaneous bacterial peritonitis and hepatic encephalopathy in the combination treatment group were significantly lower than those in the control group (spontaneous bacterial peritonitis: 0% vs 20%; *P* = 0.028; hepatic encephalopathy: 0% vs 20%; *P* = 0.028), no significant adverse events, side effects, or complications were reported in the other 7 studies [40, 42, 43, 45–48]. Based on this analysis, adult stem cell transplantation is concluded to be safe.

## Discussion

Presumably, this is the first systematic review and meta-analysis to evaluate the efficacy of innovative treatment regimen of adult stem cell transplantation combined with conventional medicine on the liver function and therapeutic outcomes of patients with ESLD. Currently, the symptomatic treatment for ESLD comprises the usage of antiviral medicine, anti-fibrosis medicine, and albumin supplementation [49]. The results from this review indicate that a treatment regimen of adult stem cell transplantation combined with traditional medicine might further delay the progression of the disease (compared to traditional medicine therapy alone). This was mainly reflected by the TBIL, ALT, AST, and ALB levels, PTA, and PT. Another important finding is the prominent difference in the MELD and CTP scores between the combination therapy and the conventional treatment regimen, suggesting a favorable therapeutic effect and clinical significance with respect to prolonging the survival of patients receiving combination therapy. This meta-analysis was designed to explore the optimal treatment regimen using adult stem cells for patients with ESLD. The results suggest that in the short and medium term, combination therapy can improve the liver function in patients with viral and alcoholic ESLD, but not in patients with ESLD caused by other etiologies, such as immune liver disease or genetic metabolic liver disease. The available evidences from results do not support the better efficacy of G-CSF mobilized stem cells or multiple injections of stem cells. Thus, this meta-analysis might provide new hypotheses and directions for designing clinical trial protocols for ESLD in the future.

Several studies have solely focused on the efficacy of interventions while they lack a description of the possible relationships between the follow-up duration and efficacy. This study fills an important knowledge gap by inferring that the efficacy of adult stem cell transplantation in combination with conventional medicine for ESLD did not increase over time (ALB being the exception). This result is consistent with the findings of Chen et al. [50], i.e., in studies with a follow-up time longer than 24 weeks, only a notable increase in the level of ALB was observed. In contrast, based on the results for most of the indicators, i.e., TBIL, ALT, AST, and CTP, changes in therapeutic effect demonstrated a quadratic trajectory that increased and reached a peak 4 weeks after stem cell transplantation followed by a gradual decline. This is consistent with the findings of Kwak et al. [51] that the improvement in liver function decreases over time after injection with ABMMSCs. These results may provide insights into the optimal timing for stem cell injections.

Due to the heterogeneity between the studied groups, a subgroup analysis was performed based on the number of adult stem cell injections, adult stem cell species, and the usage of G-CSF. An unexpected finding was that a single injection of adult stem cells had beneficial effects on TBIL and ALB levels and the MELD scores, whereas no significant improvements were observed in other groups. This finding is supported by Zhao et al. [52]. However, no prominent differences were observed in the ALT and AST levels between the two groups, which indicates that there is no significant difference in the transplantation effects based on the number of adult stem cell injections. The difference in these indicators may be attributed to the limited number of patients enrolled for receiving two injections of adult stem cells. However, in two clinical studies [44, 47] a possible reason speculated was the very short interval between the stem cell injections, i.e., the patients were unable to tolerate large doses of stem cells in a short period, thereby resulting in poor efficacy. With respect to the TBIL level, MNCs have a better therapeutic effect on patients with ESLD than ABMMSCs. However, the therapeutic effect of CD34^+^ APBSCs on ALB levels is similar to that of MNCs. The limited number of studies and outcomes made it difficult to clarify the relationship between adult stem cell species and outcomes. Moreover, the use of G-CSF, a stem cell mobilization agent, did not improve liver function in patients with ESLD.

For hepatic disorders, several studies have suggested therapy using MSCs via paracrine factors, such as IL-10, IL-1ra, and TGF-β. MSCs can inhibit excessive activation and proliferation of natural killer (NK) cells [53], which otherwise can lead to increased serum IFN-γ levels and impaired liver regeneration [44, 54]. Furthermore, MSCs significantly increase the of Treg cell counts through prostaglandin E2 (PGE2) secreted via a PI3K-dependent pathway that effectively reduces immune response‒mediated liver injury [44, 55, 56]. In addition, MSCs can exhibit an anti-inflammatory effect by inducing a shift in macrophage polarization from the M1–M2 phenotype [57, 58]. Thus, the immunomodulatory function of MSCs can be summarized as an attenuation of the pro-inflammatory response and enhancement of the anti-inflammatory response that promotes the immune environment homeostasis and encourages liver repair.

There are a few limitations to this study. First, as the number of studies and patients whose data were included in the clinical studies was relatively small, it was difficult to stratify for potential confounders—such as age—that are closely associated with hepatic function and severe complications [59, 60]. Second, as the outcome indicators and duration of follow-up were not consistent in the studies, only a limited number of studies were pooled to assess certain outcomes. Third, as the hepatic artery route was used in seven [40–43, 45–47] of the eight studies, subgroup analyses were not conducted according to the types of routes of adult stem cell transplantation. Thus, the results from this study investigating the efficacy of a combination of adult stem cell transplantation and traditional medicine in patients with ESLD should be interpreted with caution.

## Conclusions

**Fig. 8:**
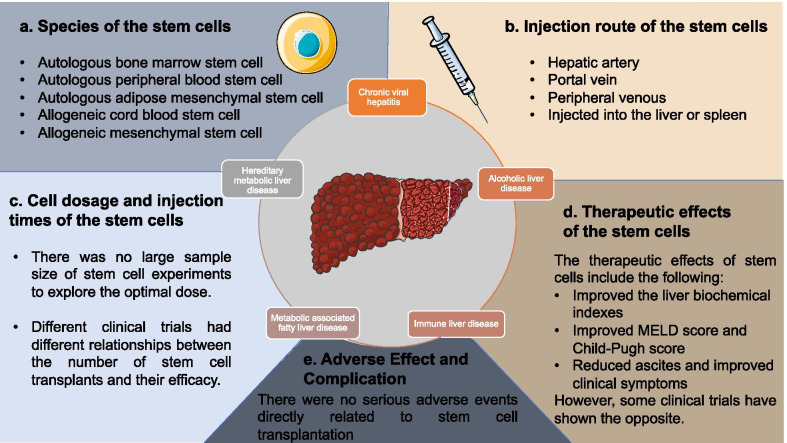
A summary of current studies for stem cells in treating ESLD. ESLD can divide into five diseases on the circle based on etiology. Among them, the clinical evidence for chronic viral hepatitis and alcoholic liver disease is relatively sufficient. **A** The optimal type of stem cell for the treatment of ESLD is still unclear. **B** There is not enough evidence for the difference of therapeutic effect between different injection routes of stem cells in the treatment of ESLD. **C** The dose–response relationship and times of injection of stem cells remain to be studied. **D** Most studies have shown superior therapeutic effects from stem cells, but the benefit is short, and the existence of negative results makes conclusions controversial. **E** Most studies had a short follow-up time after stem cell transplantation and might not have adverse effects. Some illustrations were produced using Servier Medical Art. https://smart.servier.com

The present systematic review and meta-analysis indicates that with respect to the improvement in liver function and therapeutic outcomes for patients with ESLD, the benefits of adult stem cells combined with conventional medicine were slightly better than those of the traditional treatment. However, this efficacy gradually declined with the extension of the follow-up duration. Further, high-quality and well-designed randomized controlled trials (RCTs) are needed with larger samples to determine the optimal route, dose, and timing of adult stem cell transplantation in patients with ESLD (Fig. 8).

## Supplementary Information

**Additional file 1:** **Fig. S1.** Forest plot of PTA at different time points with the combination therapy compared with traditional therapy. Compared with the traditional therapy group, PTA in the combination therapy group was significantly higher at 12, 24, 36, and 48 weeks (*P* < 0.05). No significant heterogeneity was observed, and a fixedeffects model was used for statistical analysis. In the plane rectangular coordinate system, the forest plot takes a vertical invalid line (scale of abscissa is 0) as the center, describes the effect quantity and 95% CI of each study by using multiple line segments parallel to the horizontal axis, and describes the effect quantity and confidence interval of multiple studies by using a diamond. *PTA*, prothrombin time activity; *CI*, confidence interval; *WMD*, weighted mean difference. **Fig. S2.** Forest plot of PT at different time points with the combination therapy compared with traditional therapy. Compared with the traditional therapy group, PT in the combination therapy group was nonsignificantly lower at 1 week (*P* > 0.05), significantly lower at 2, 4, 12, 24, and 48 weeks (*P* < 0.05). Since significant heterogeneity was observed in one group (I2 = 87%), a random-effects model was used. In the plane rectangular coordinate system, the forest plot takes a vertical invalid line (scale of abscissa is 0) as the center, describes the effect quantity and 95% CI of each study by using multiple line segments parallel to the horizontal axis, and describes the effect quantity and confidence interval of multiple studies by using a diamond. *PT*, prothrombin time; *CI*, confidence interval. *WMD*, weighted mean difference. **Fig. S3.** Forest plot of the subgroup analysis of the 6-month clinical performance according to the number of stem cell injection cycles. (**A**) TBIL; (**B**) ALB; (**C**) ALT. The level of TBIL, and ALB in patients who received single-injection of stem cells were significantly different as compared to those in the patients who received two-injections of stem cells (*P* < 0.05), nonsignificantly difference for the level of ALT (*P* > 0.05). No significant heterogeneity was observed, and a fixed-effects model was used for statistical analysis. In the plane rectangular coordinate system, the forest plot takes a vertical invalid line (scale of abscissa is 0) as the center, describes the effect quantity and 95% CI of each study by using multiple line segments parallel to the horizontal axis, and describes the effect quantity and confidence interval of multiple studies by using a diamond.* TBIL*, total bilirubin;* ALB*, albumin;* ALT*, alanine aminotransferase;* CI*, confidence interval;* SMD*, standardized mean difference. **Fig. S4.** Forest plot of the subgroup analysis of the 6-month clinical performance according to the number of stem cell injection cycles. (**A**) AST; (**B**) CTP; (**C**) MELD. The level of CTP, and MELD in patients who received single-injection of stem cells were significantly different as compared to those in the patients who received two-injections of stem cells (*P* < 0.05), nonsignificantly difference for the level of AST (*P* > 0.05). Since no significant heterogeneity was observed in the figure A and B, a fixed-effect model was used. However, significant heterogeneity was observed in figure C (I2 = 85%), therefore, a random-effects model was used. In the plane rectangular coordinate system, the forest plot takes a vertical invalid line (scale of abscissa is 0) as the center, describes the effect quantity and 95% CI of each study by using multiple line segments parallel to the horizontal axis, and describes the effect quantity and confidence interval of multiple studies by using a diamond.* AST*, aspartate aminotransferase;* CTP*, Child-Pugh score;* MELD*, Model for end-stage liver disease;* CI*, confidence interval;* SMD*, standardized mean difference. **Fig. S5.** Forest plot of the subgroup analysis of the 6-month clinical performance according to the species of adult stem cells. (**A**) TBIL; (**B**) ALB. The level of TBIL decreased considerably in the studies transplanting MNCs (*P* < 0.05), nonsignificantly different in the studies transplanting CD34 + APBSC or ABMMSC (*P*>0.05). In addition, the level of ALB in patients who received CD34 + APBSC or MNCs were significantly different (*P* < 0.05), nonsignificantly different in patients who received ABMMSC (*P* > 0.05). No significant heterogeneity was observed, and a fixed-effects model was used for statistical analysis. In the plane rectangular coordinate system, the forest plot takes a vertical invalid line (scale of abscissa is 0) as the center, describes the effect quantity and 95% CI of each study by using multiple line segments parallel to the horizontal axis, and describes the effect quantity and confidence interval of multiple studies by using a diamond.* TBIL*, total bilirubin;* ALB*, albumin;* CI*, confidence interval;* SMD*, standardized mean difference. **Fig. S6.** Forest plot of subgroup analysis. (**A**) Subgroup analysis of the use of G-CSF in terms of TBIL at twelve weeks. (**B**) Subgroup analysis of the use of G-CSF in terms of TBIL at twenty-four weeks. (**C**) Subgroup analysis of the use of G-CSF in terms of ALB at twelve weeks. (**D**) Subgroup analysis of the use of GCSF in terms of ALB at twenty-four weeks. The level of TBIL at 12, and 24 weeks in patients who received G-CSF mobilized stem cells were nonsignificantly different (*P* > 0.05). The rest of the subgroups were significantly different (*P* < 0.05). No significant heterogeneity was observed, and a fixed-effects model was used for statistical analysis. In the plane rectangular coordinate system, the forest plot takes a vertical invalid line (scale of abscissa is 0) as the center, describes the effect quantity and 95% CI of each study by using multiple line segments parallel to the horizontal axis, and describes the effect quantity and confidence interval of multiple studies by using a diamond.* TBIL*, total bilirubin;* ALB*, albumin;* G-CSF*, granulocyte colony-stimulating factor;* CI*, confidence interval;* SMD*, standardized mean difference. **Fig. S7.** Forest plot of subgroup analysis. (**A**) Subgroup analysis of the use of G-CSF in terms of ALT at twelve weeks. (**B**) Subgroup analysis of the use of G-CSF in terms of ALT at twenty-four weeks. (**C**) Subgroup analysis of the use of G-CSF in terms of AST at twenty-four weeks. The level of ALT at 12 weeks in patients who received stem cells collected directly were significantly different (*P* < 0.05). The rest of the subgroups were nonsignificantly different (*P* > 0.05). No significant heterogeneity was observed in the figure B, and C, therefore, a fixed-effect model was used. Since significant heterogeneity was observed in figure A (I2 = 89%), a random-effects model rather was used. In the plane rectangular coordinate system, the forest plot takes a vertical invalid line (scale of abscissa is 0) as the center, describes the effect quantity and 95% CI of each study by using multiple line segments parallel to the horizontal axis, and describes the effect quantity and confidence interval of multiple studies by using a diamond.* ALT*: alanine aminotransferase;* AST*: aspartate aminotransferase;* G-CSF*, granulocyte colony-stimulating factor;* CI*, confidence interval;* SMD*, standardized mean difference.

## Data Availability

N/A.

## Abbreviations

ESLD: End-stage liver disease
MELD: Model for end-stage liver disease
CTP: Child‒Turcotte‒Pugh
TBIL: Total bilirubin
ALB: Albumin
AST: Aspartate aminotransferase
ALT: Alanine aminotransferase
MNCs: Mononuclear cells
MSCs: Mesenchymal stromal cells
NK: Natural killer
PGE2: Prostaglandin E2
CD34^+^ APBSC: CD34^+^ autologous peripheral blood stem cell
ICU: Intensive care unit
BMT: Bone marrow transplantation
HSC/Ps: Hematopoietic stem cell/progenitors
CD34^+^ PBSCs: CD34^+^ peripheral blood stem cells
BMMSCs: Bone marrow-derived mesenchymal stromal cells
BMMNCs: Bone marrow mononuclear cells
RCTs: Randomized controlled trials
MeSH: Medical Subject Headings
PT: Prothrombin time
PTA: Prothrombin time activity
SMD: Standardized mean difference
WMD: Weighted mean difference
CI: Confidence interval
Q test: Chi-square test
HCV: Hepatitis C virus (HCV)
HBV: Hepatitis B virus
G-CSF: Granulocyte colony-stimulating factor
