# Supplementary material for: Adult stem cell transplantation combined with conventional therapy for the treatment of end-stage liver disease: a systematic review and meta-analysis
Source: Stem Cell Res Ther. 2021 Oct 30;12:558. doi: 10.1186/s13287-021-02625-x (PMC8557537; doi:10.1186/s13287-021-02625-x)
Supplement: Supplementary file 1 — Additional file 1: Fig. S1. Forest plot of PTA at different time points with the combination therapy compared with traditional therapy. Compared with the traditional therapy group, PTA in the combination therapy group was significantly higher at 12, 24, 36, and 48 weeks (P < 0.05). No significant heterogeneity was observed, and a fixedeffects model was used for statistical analysis. In the plane rectangular coordinate system, the forest plot takes a vertical invalid line (scale of abscissa is 0) as the center, describes the effect quantity and 95% CI of each study by using multiple line segments parallel to the horizontal axis, and describes the effect quantity and confidence interval of multiple studies by using a diamond. PTA, prothrombin time activity; CI, confidence interval; WMD, weighted mean difference. Fig. S2. Forest plot of PT at different time points with the combination therapy compared with traditional therapy. Compared with the traditional therapy group, PT in the combination therapy group was nonsignificantly lower at 1 week (P > 0.05), significantly lower at 2, 4, 12, 24, and 48 weeks (P < 0.05). Since significant heterogeneity was observed in one group (I2 = 87%), a random-effects model was used. In the plane rectangular coordinate system, the forest plot takes a vertical invalid line (scale of abscissa is 0) as the center, describes the effect quantity and 95% CI of each study by using multiple line segments parallel to the horizontal axis, and describes the effect quantity and confidence interval of multiple studies by using a diamond. PT, prothrombin time; CI, confidence interval. WMD, weighted mean difference. Fig. S3. Forest plot of the subgroup analysis of the 6-month clinical performance according to the number of stem cell injection cycles. (A) TBIL; (B) ALB; (C) ALT. The level of TBIL, and ALB in patients who received single-injection of stem cells were significantly different as compared to those in the patients who received two-injections [file 13287_2021_2625_MOESM1_ESM.docx]

**Additional Files**

**Additional file 1: Fig. S1** Forest plot of PTA at different time points with the combination therapy compared with traditional therapy. Compared with the traditional therapy group, PTA in the combination therapy group was significantly higher at 12, 24, 36, and 48 weeks (P<0.05). No significant heterogeneity was observed, and a fixed-effects model was used for statistical analysis. In the plane rectangular coordinate system, the forest plot takes a vertical invalid line (scale of abscissa is 0) as the center, describes the effect quantity and 95% CI of each study by using multiple line segments parallel to the horizontal axis, and describes the effect quantity and confidence interval of multiple studies by using a diamond. PTA, prothrombin time activity; CI, confidence interval; WMD, weighted mean difference.

**Additional file 2: Fig. S2** Forest plot of PT at different time points with the combination therapy compared with traditional therapy. Compared with the traditional therapy group, PT in the combination therapy group was nonsignificantly lower at 1 week (P>0.05), significantly lower at 2, 4, 12, 24, and 48 weeks (P<0.05). Since significant heterogeneity was observed in one group (I^2^=87%), a random-effects model was used. In the plane rectangular coordinate system, the forest plot takes a vertical invalid line (scale of abscissa is 0) as the center, describes the effect quantity and 95% CI of each study by using multiple line segments parallel to the horizontal axis, and describes the effect quantity and confidence interval of multiple studies by using a diamond. PT, prothrombin time; CI, confidence interval. WMD, weighted mean difference.

**Additional file 3: Fig. S3** Forest plot of the subgroup analysis of the 6-month clinical performance according to the number of stem cell injection cycles. (A) TBIL; (B) ALB; (C) ALT. The level of TBIL, and ALB in patients who received single-injection of stem cells were significantly different as compared to those in the patients who received two-injections of stem cells (P<0.05), nonsignificantly difference for the level of ALT (P>0.05). No significant heterogeneity was observed, and a fixed-effects model was used for statistical analysis. In the plane rectangular coordinate system, the forest plot takes a vertical invalid line (scale of abscissa is 0) as the center, describes the effect quantity and 95% CI of each study by using multiple line segments parallel to the horizontal axis, and describes the effect quantity and confidence interval of multiple studies by using a diamond. TBIL, total bilirubin; ALB, albumin; ALT, alanine aminotransferase; CI, confidence interval; SMD, standardized mean difference.

**Additional file 4: Fig. S4** Forest plot of the subgroup analysis of the 6-month clinical performance according to the number of stem cell injection cycles. (A) AST; (B) CTP; (C) MELD. The level of CTP, and MELD in patients who received single-injection of stem cells were significantly different as compared to those in the patients who received two-injections of stem cells (P<0.05), nonsignificantly difference for the level of AST (P>0.05). Since no significant heterogeneity was observed in the figure A and B, a fixed-effect model was used. However, significant heterogeneity was observed in figure C (I^2^=85%), therefore, a random-effects model was used. In the plane rectangular coordinate system, the forest plot takes a vertical invalid line (scale of abscissa is 0) as the center, describes the effect quantity and 95% CI of each study by using multiple line segments parallel to the horizontal axis, and describes the effect quantity and confidence interval of multiple studies by using a diamond. AST, aspartate aminotransferase; CTP, Child-Pugh score; MELD, Model for end-stage liver disease; CI, confidence interval; SMD, standardized mean difference.

**Additional file 5: Fig. S5** Forest plot of the subgroup analysis of the 6-month clinical performance according to the species of adult stem cells. (A) TBIL; (B) ALB. The level of TBIL decreased considerably in the studies transplanting MNCs (P<0.05), nonsignificantly different in the studies transplanting CD34^+^APBSC or ABMMSC (P>0.05). In addition, the level of ALB in patients who received CD34^+^APBSC or MNCs were significantly different (P<0.05), nonsignificantly different in patients who received ABMMSC (P>0.05). No significant heterogeneity was observed, and a fixed-effects model was used for statistical analysis. In the plane rectangular coordinate system, the forest plot takes a vertical invalid line (scale of abscissa is 0) as the center, describes the effect quantity and 95% CI of each study by using multiple line segments parallel to the horizontal axis, and describes the effect quantity and confidence interval of multiple studies by using a diamond. TBIL, total bilirubin; ALB, albumin; CI, confidence interval; SMD, standardized mean difference.

**Additional file 6: Fig. S6** Forest plot of subgroup analysis. (A) Subgroup analysis of the use of G-CSF in terms of TBIL at twelve weeks. (B) Subgroup analysis of the use of G-CSF in terms of TBIL at twenty-four weeks. (C) Subgroup analysis of the use of G-CSF in terms of ALB at twelve weeks. (D) Subgroup analysis of the use of G-CSF in terms of ALB at twenty-four weeks. The level of TBIL at 12, and 24 weeks in patients who received G-CSF mobilized stem cells were nonsignificantly different (P>0.05). The rest of the subgroups were significantly different (P<0.05). No significant heterogeneity was observed, and a fixed-effects model was used for statistical analysis. In the plane rectangular coordinate system, the forest plot takes a vertical invalid line (scale of abscissa is 0) as the center, describes the effect quantity and 95% CI of each study by using multiple line segments parallel to the horizontal axis, and describes the effect quantity and confidence interval of multiple studies by using a diamond. TBIL, total bilirubin; ALB, albumin; G-CSF, granulocyte colony-stimulating factor; CI, confidence interval; SMD, standardized mean difference.

**Additional file 7: Fig. S7** Forest plot of subgroup analysis. (A) Subgroup analysis of the use of G-CSF in terms of ALT at twelve weeks. (B) Subgroup analysis of the use of G-CSF in terms of ALT at twenty-four weeks. (C) Subgroup analysis of the use of G-CSF in terms of AST at twenty-four weeks. The level of ALT at 12 weeks in patients who received stem cells collected directly were significantly different (P<0.05). The rest of the subgroups were nonsignificantly different (P>0.05). No significant heterogeneity was observed in the figure B, and C, therefore, a fixed-effect model was used. Since significant heterogeneity was observed in figure A (I^2^=89%), a random-effects model rather was used. In the plane rectangular coordinate system, the forest plot takes a vertical invalid line (scale of abscissa is 0) as the center, describes the effect quantity and 95% CI of each study by using multiple line segments parallel to the horizontal axis, and describes the effect quantity and confidence interval of multiple studies by using a diamond. ALT: alanine aminotransferase; AST: aspartate aminotransferase; G-CSF, granulocyte colony-stimulating factor; CI, confidence interval; SMD, standardized mean difference.

Fig.S1

Fig.S2

Fig.S3

Fig.S4

Fig.S5

Fig.S6

Fig.S7
